# Trichophyton tonsurans infections after visiting a barbershop – findings from official hygiene monitoring

**DOI:** 10.3205/dgkh000507

**Published:** 2024-10-23

**Authors:** Anne Marcic, Stephen Freytag, Katharina Langen

**Affiliations:** 1 Public Health Department of the State Capital Kiel, Infection Protection Department, Kiel, Germany; 2 University Hospital Schleswig-Holstein, Campus Kiel, Clinic for Dermatology, Venereology and Allergology, Kiel, Germany

**Keywords:** Trichophyton tonsurans, barbershop, disinfection measures, reprocessing of work equipment, official hygiene monitoring

## Abstract

**Background:**

The Public Health Department became aware of infections with *Trichophyton (T.) tonsurans* in a total of nine people who had visited the same barbershop.

**Method:**

Official hygiene monitoring of the barbershop was performed on an event-related basis, during which compliance with the requirements of the “Schleswig-Holstein State Ordinance on the Prevention of Communicable Diseases (Hygiene Ordinance)” was checked. In addition, hygienic-microbiological environmental examinations of materials and surfaces were performed in cooperation with the Department of Dermatology at the University Medical Center Schleswig-Holstein, Kiel Campus.

**Results:**

Significant deficiencies in hygiene management were identified. The hygiene requirements, especially for the reprocessing of work equipment and surfaces, were not known and were therefore not complied with. Environmental testing revealed the presence of *Trichophyton tonsurans* in three out of ten samples tested. Shaving equipment and a drawer used to store shaving equipment were positive.

**Conclusion:**

Barbershops can pose a relevant risk of infection, not only for pathogens that cause blood-borne infections. Barbershop operators need information and training regarding compliance with hygiene requirements. They should be included in the planning for the (random) infection hygiene monitoring of facilities by the public health department in accordance with Section 36 (2) of the Infection Protection Act (IfSG). The reprocessing frequencies established to date are not sufficient for the prevention of *T. tonsurans* infections and must be adapted.

## Zusammenfassung

**Hintergrund:**

Dem Amt für Gesundheit wurden Infektionen mit *Trichophyton (T.) tonsurans* bei neun Personen bekannt, die denselben Barbershop besucht hatten.

**Methode:**

Es fand eine anlassbezogene infektionshygienische Überwachung des Barbershops statt, bei der die Einhaltung der Anforderungen nach der „Landesverordnung zur Verhütung übertragbarer Krankheiten Schleswig-Holstein (HygieneVO)“ überprüft wurde. Zusätzlich wurden in Zusammenarbeit mit der Klinik für Dermatologie des Universitätsklinikums Schleswig-Holstein, Campus Kiel, hygienisch-mikrobiologische Umgebungsuntersuchungen von Materialien und Oberflächen durchgeführt.

**Ergebnisse:**

Es wurden deutliche Mängel im Hygienemanagement festgestellt. Die Anforderungen an die Hygiene, insbesondere an die Aufbereitung von Arbeitsgeräten und Flächen, waren nicht bekannt und wurden demzufolge nicht eingehalten. Die Umgebungsuntersuchung ergab den Nachweis von *T. tonsurans* in drei von zehn untersuchten Proben. Positiv waren Rasiergeräte und eine Schublade, die zur Lagerung von Rasiergeräten genutzt wurde.

**Schlussfolgerung:**

Von Barbershops kann eine relevante Infektionsgefahr nicht nur für Erreger blutübertragbarer Infektionen ausgehen. Bei Betreibern von Barbershops besteht ein Informations- und Schulungsbedarf im Hinblick auf die Einhaltung der Anforderungen an die Hygiene. Sie sollten in die Planung für die (stichprobenartige) infektionshygienische Überwachung von Einrichtungen nach § 36 Absatz 2 Infektionsschutzgesetz (IfSG) durch das Gesundheitsamt einbezogen werden. Die bisher etablierten Aufbereitungsfrequenzen sind für die Prävention von* T. tonsurans*-Infektionen nicht ausreichend und müssen angepasst werden.

## Introduction

In July 2023, the Office of Public Health received a complaint from a citizen about a fungal infection on the back of his head after visiting a barbershop (Barbershop A).

Barbershops carry out activities on humans in which pathogens, in particular blood-borne pathogens, can be transmitted to humans. They are therefore subject to the “Schleswig-Holstein State Ordinance on the Prevention of Communicable Diseases (Hygiene Ordinance)” and must draw up a hygiene plan in accordance with Section 2 (6) of the Hygiene Ordinance, in which all internal infection hygiene procedures are recorded in writing [1]. They are among the facilities that can be monitored for infection hygiene in accordance with Section 36 (2) of the Infection Protection Act (IfSG) [2].

During the investigations, further fungal infections were discovered in connection with visits to the same barbershop. Pathogen detection was not yet available at this time but was subsequently found in other patients who presented at the dermatology clinic at the university hospital.

Initially, there was an event-related infection hygiene monitoring of Barbershop A, which was followed by further on-site visits to check hygiene management and the reprocessing of the materials used. In addition, hygienic-microbiological environmental examinations were performed. The situation gave rise to the monitoring of further barbershops and the derivation of consequences regarding information and training as well as monitoring planning.

## Materials and methods

As part of an event-related inspection, the requirements of the Hygiene Ordinance for the prevention of communicable diseases were checked, including hand hygiene, reprocessing of work materials (razors, combs, scissors), surface cleaning and disinfection, reprocessing of textiles (towels and capes) and waste disposal. 

**Figure 1:**
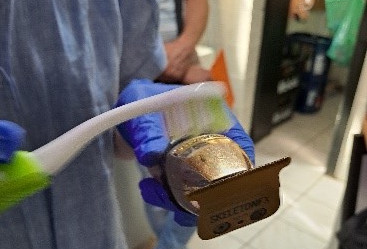
Sampling using brushes

**Figure 2:**
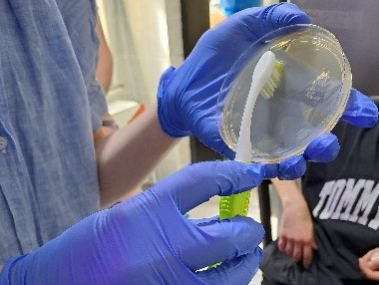
Imprint on dermatophyte selective agar plates

**Figure 3:**
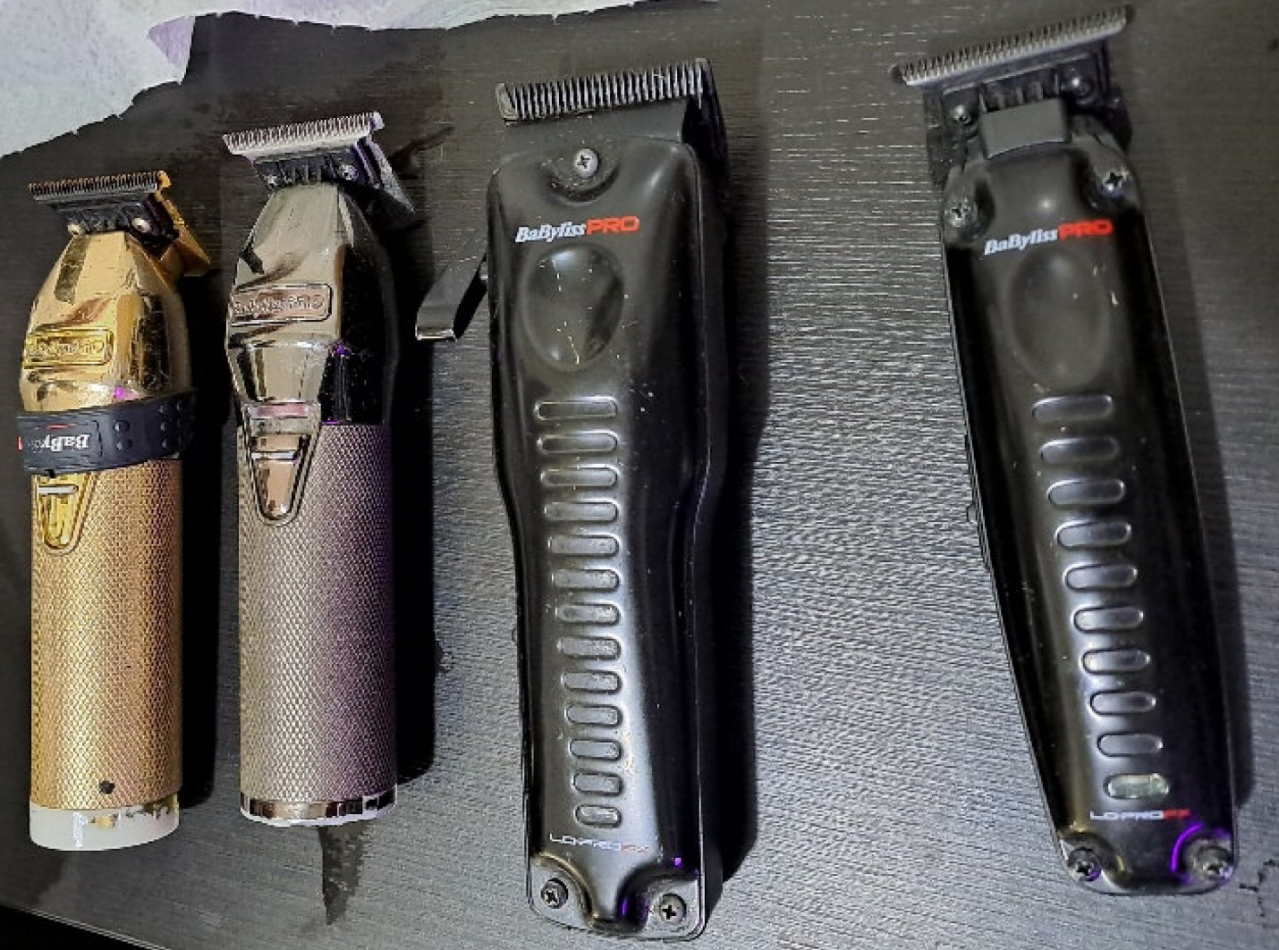
Razors for hygienic-microbiological examination

In the further course, a hygienic-microbiological environmental examination was performed at a follow-up appointment in cooperation with the Department of Dermatology at the University Hospital Schleswig-Holstein, Kiel Campus. Samples were taken from surfaces using brushes and imprints on dermatophyte-selective agar plates (with cycloheximide and gentamicin) (Figure 1 (Fig. 1) and Figure 2 (Fig. 2)). Surfaces and devices were selected for examination that were possible sources of infection, including storage surface for razors, drawer for razors, four different razors (Figure 3 (Fig. 3)) and the head washbasin. The selective agar plates were incubated at 27°C for 28 days.

## Results

**Figure 4:**
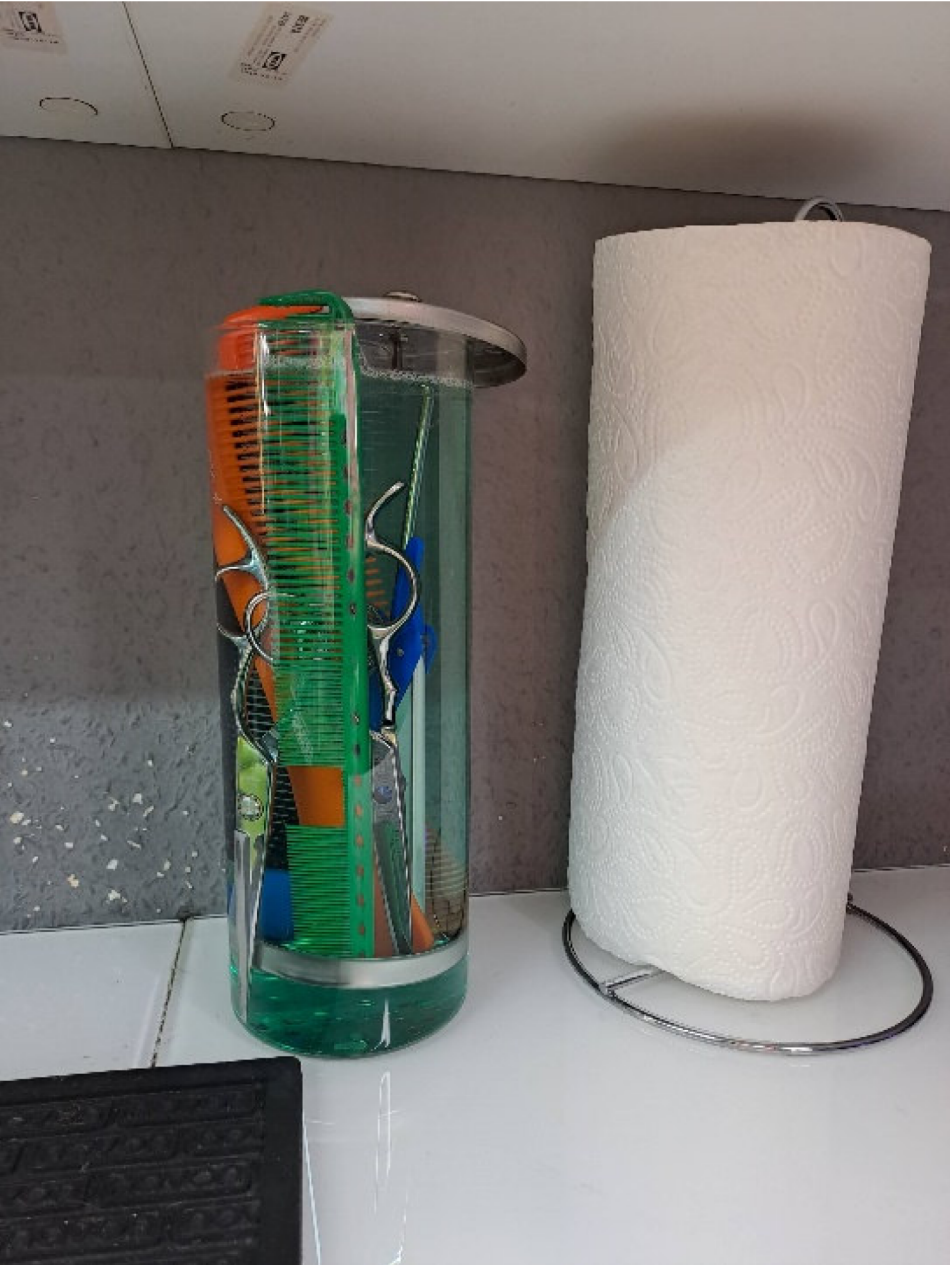
Device reprocessing at the time of the first inspection

Barbershop A was not aware of the requirements of the Hygiene Ordinance, including the requirement to use VAH-listed disinfectants. No hygiene plan was available. Some of the work materials were reprocessed with an aldehyde-based instrument disinfectant in very high concentrations without completely wetting the materials to be reprocessed in a glass container commonly used in barbershops (Figure 4 (Fig. 4)). The surfaces and some work materials were treated with commercially available drugstore products and additionally with special cleaning agents. Towels were washed at 40°C and machine dried.

**Figure 5:**
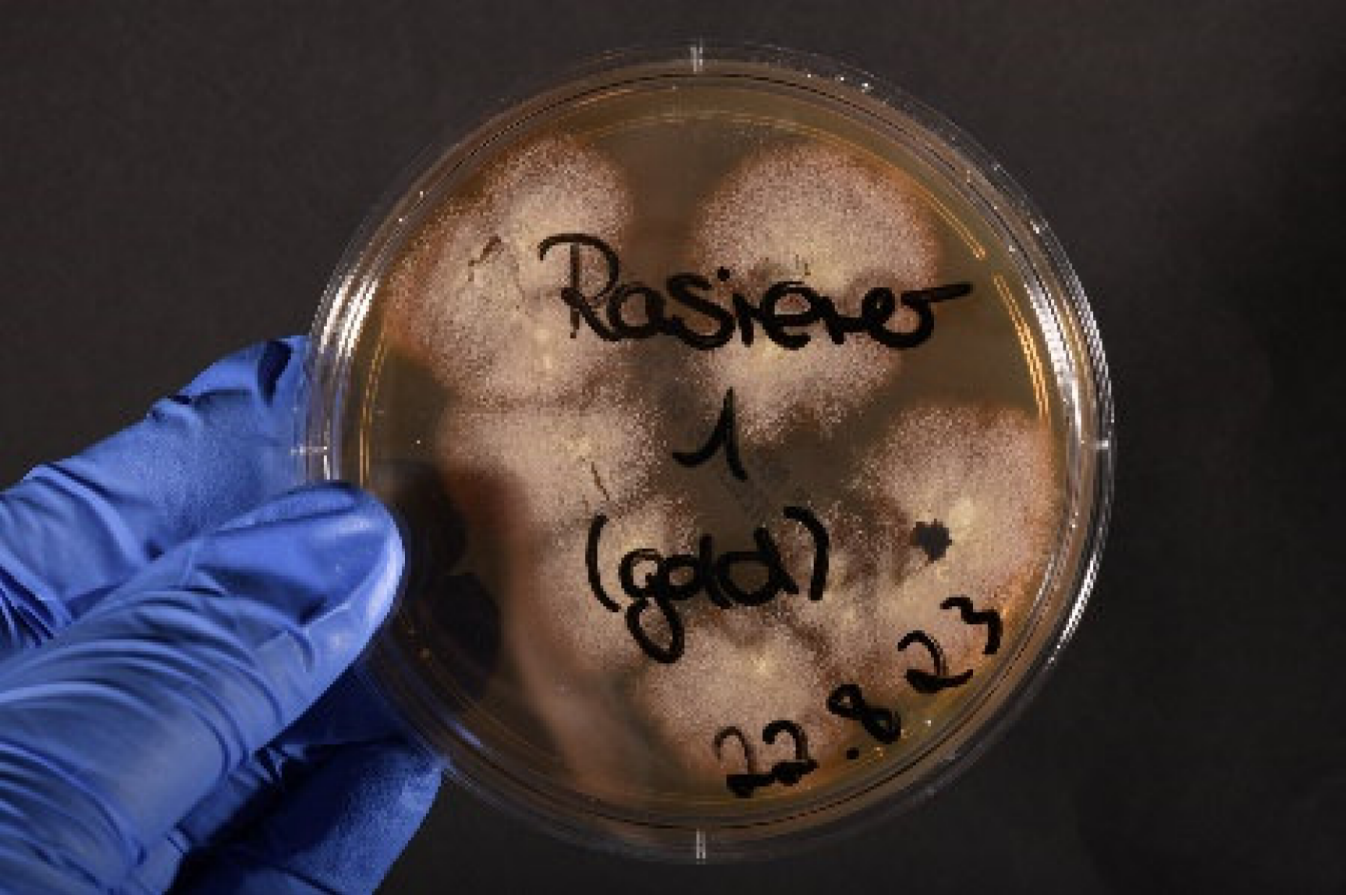
Growth of *T. tonsurans*, imprint of a razor

**Figure 6:**
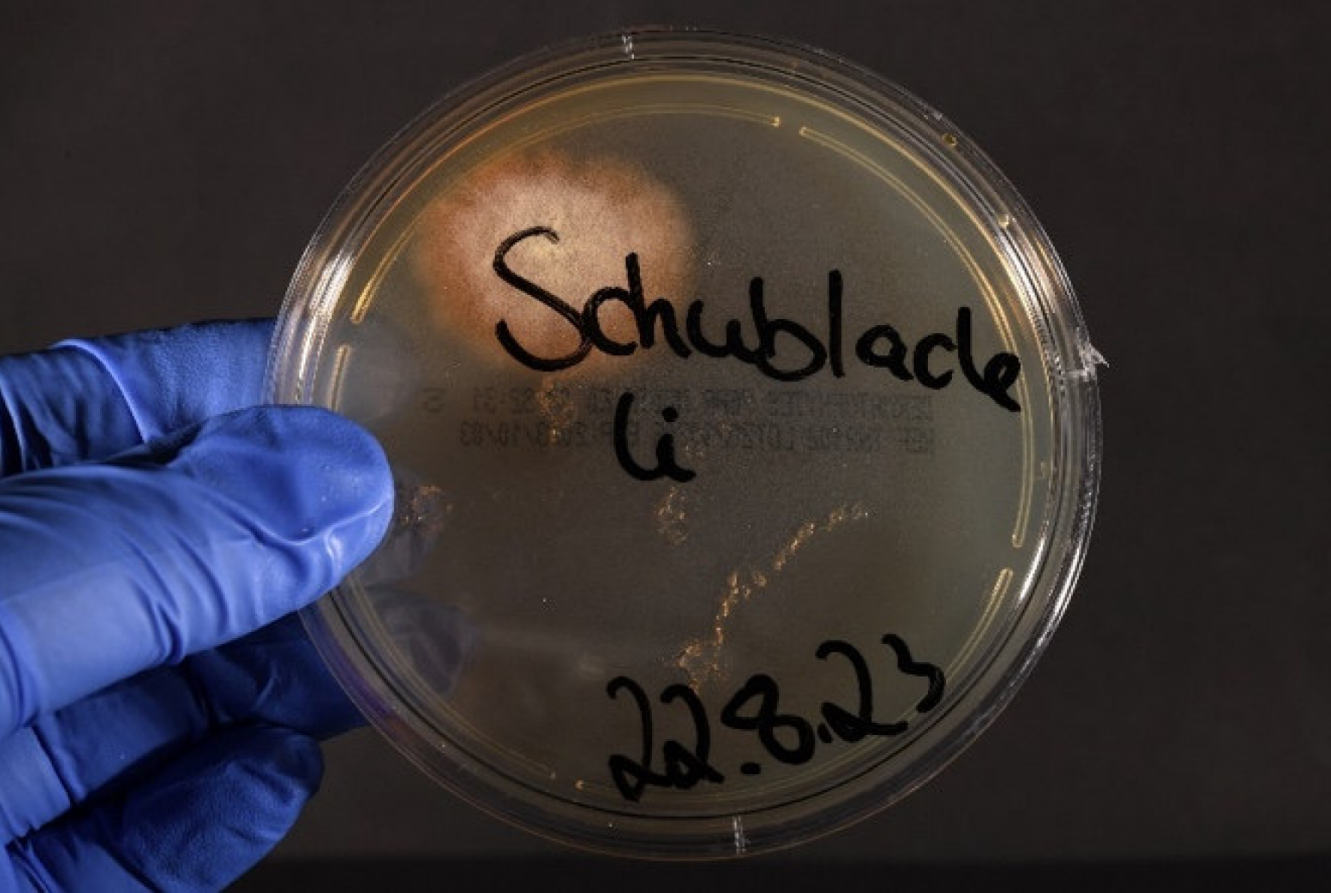
Growth of T. tonsurans, drawer

Hygienic microbiological sampling in Barbershop A revealed the presence of *T. tonsurans* in three out of ten samples tested. The detection was carried out on two razors, one of which showed pronounced growth (Figure 5 (Fig. 5)), as well as in the drawer in which razors were stored (Figure 6 (Fig. 6)).

**Figure 7:**
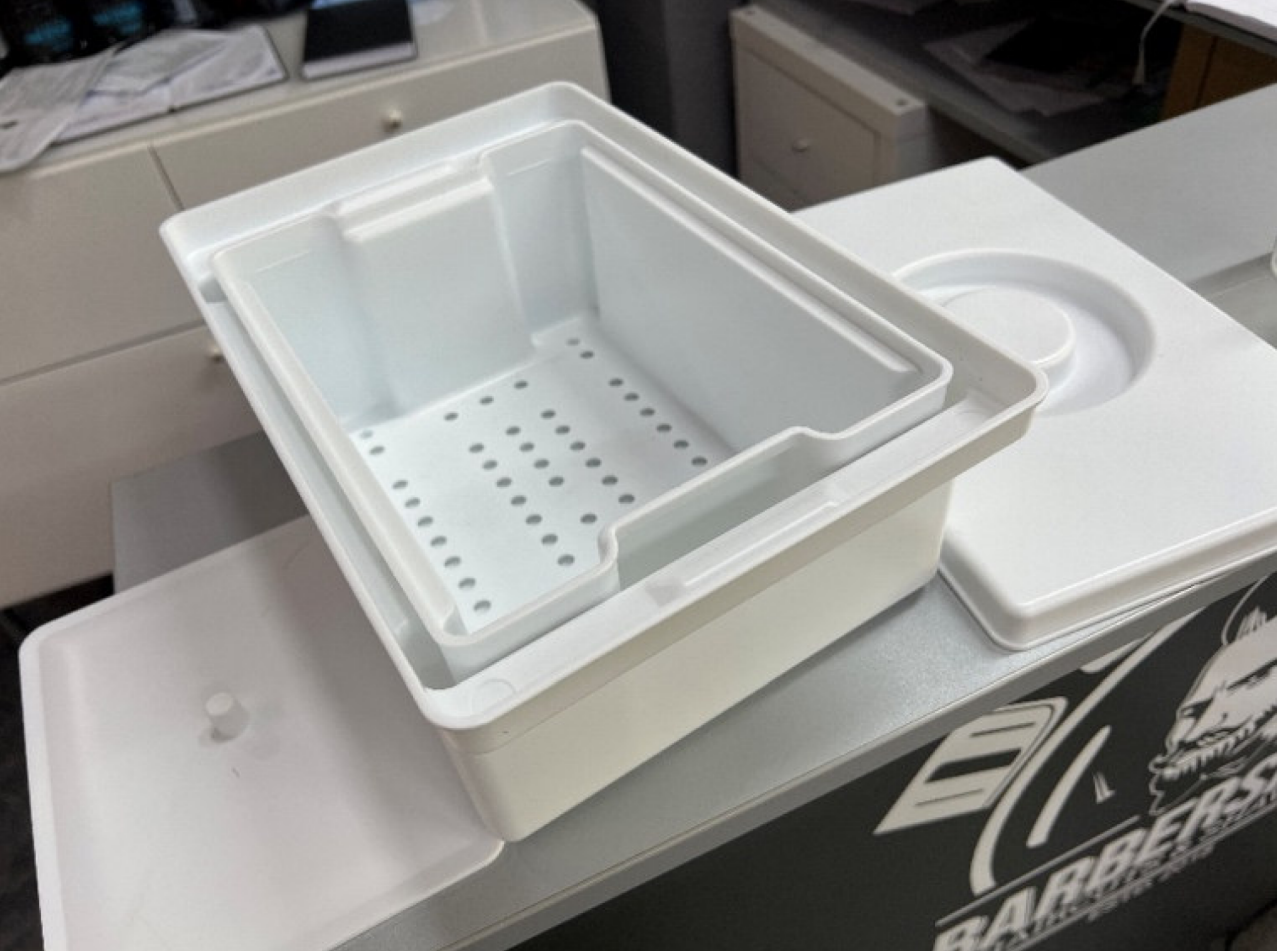
Newly purchased instrument disinfection tray

The elimination of the hygiene deficiencies was checked during further site inspections. Information materials on the requirements for hygiene measures in accordance with the Hygiene Ordinance and the creation of a hygiene plan were handed out and the conversion and establishment of proper reprocessing of work materials was accompanied (Figure 7 (Fig. 7))

In our experience, barbershops generally do not receive sufficient information on which to establish the necessary hygiene measures when registering their business and commencing operations. For this reason, an information package was created that the trade supervisory authority can hand out when registering a business.

Routine sample hygiene plans for the hairdressing trade provide for the daily reprocessing of working materials and reprocessing after visible contamination with blood and secretions [3]. This is not sufficient to prevent the transmission of *T. tonsurans*. As a consequence, a leaflet for addressees of the Hygiene Ordinance was created in which the routine disinfection of work materials was defined and supplemented by the addition “after contact with customers with suspected skin infection” [4].

## Discussion

Barbershops have been described as a source of *T. tonsurans* infections [5], [6]. In Kiel, an accumulation of *T. tonsurans* infections occurred in July 2023, which initially came to the attention of the public health department through a citizen complaint. During the investigations and event-related inspections of the barbershop concerned, fundamental hygiene deficiencies were identified that were due to ignorance of the requirements. At the same time, the barbershop owner was willing to cooperate. The legal and professional requirements for businesses that carry out activities involving humans, in which pathogens can be transmitted to humans, were not known. Accordingly, gaps in hygiene management were identified which are associated with a risk of infection, particularly regarding reprocessing and surface disinfection.

*T. tonsurans* is a dermatophyte with a worldwide distribution. Transmission between people can occur directly through infectious skin flakes. In the case described, indirect transmission through work materials and surfaces was investigated in connection with a visit to a barbershop. *T. tonsurans* was detected in three environmental samples from Barbershop A. The detection on shaving equipment and in a drawer for storing shaving equipment confirms the inadequate preparation of work materials and surfaces, and underlines the need for action. 

Model hygiene plans [3] include disinfection as a requirement for the reprocessing of work materials, generally only daily and in the event of visible contamination with blood or secretions. This requirement does not do justice to the risk of infection by *T. tonsurans*. To minimize the risk of transmission, the equipment used would also have to be disinfected after contact with a customer with a suspected skin infection.

The situation gave rise to the monitoring of additional barbershops for infection hygiene.

Neither the legal regulations on hygiene requirements nor the necessary measures were known in the monitored barbershops.

Overall, there was a willingness to cooperate on the part of the barbershop owners, and a fundamental interest in complying with the requirements and eliminating potential sources of infection.

The reprocessing frequency requirements must be increased in view of the risk of infection with *T. tonsurans*. Reprocessing after customers with suspected skin infection seems appropriate.

## Conclusions

Barbershops can pose a relevant risk of infection, not only for pathogens that cause blood-borne infections. Barbershop operators require information and training regarding compliance with hygiene requirements. They should receive regular information on hygiene requirements and be included in the planning for the (random) infection hygiene monitoring of facilities by the public health department in accordance with Section 36(2) IfSG. Monitoring should focus on the reprocessing of work materials (shaving equipment) and the handling of these as well as the disinfection of relevant surfaces. The daily reprocessing frequencies established to date are not sufficient for the prevention of *T. tonsurans* infections and must be adapted so that reprocessing additionally takes place after serving clients who are suspected of having skin infections.

## Notes

### Competing interests

The authors declare that they have no competing interests.

### Author’s ORCID

- Anne Marcic: 0009-0008-3660-040X

### Supplementary note

The original version of the article was published in Hygiene & Medizin: 

Marcic A, Freytag S, Langen K. Trichophyton tonsurans-Infektionen nach Barbershop-Besuch – Erkenntnisse aus der infektionshygienischen Überwachung. HygMed. 2024;49(3):D6-D9. The original article has been minimally modified.

The topic was also presented at the 73^rd^ scientific ÖGD congress of BVÖG, BZÖG, and DGÖG and at the 17^th^ Congress for Hospital Hygiene of the German Society for General and Hospital Hygiene e.V.

## References

[1] (2007). Landesverordnung zur Verhütung übertragbarer Krankheiten (HygieneVO) vom 11. Oktober 2007.

[2] Gesetz zur Verhütung und Bekämpfung von Infektionskrankheiten beim Menschen (Infektionsschutzgesetz - IfSG) § 36 Infektionsschutz bei bestimmten Einrichtungen, Unternehmen und Personen; Verordnungsermächtigung.

[3] Berufsgenossenschaft für Gesundheitspflege und Wolhfahrtpflege (2023). Hygiene im Friseursalon Reinigungs- und Desinfektionsplan Artikelnummer BGW 06-12-090.

[4] Amt für Gesundheit der Landeshauptstadt Kiel (2023). Merkblatt für Tätigkeiten gemäß HygieneVO.

[5] Müller VL, Kappa-Markovi K, Hyun J, Georgas D, Silberfarb G, Paasch U, Uhrlaß S, Nenoff P, Schaller J (2021). Tinea capitis et barbae caused by Trichophyton tonsurans: A retrospective cohort study of an infection chain after shavings in barber shops. Mycoses.

[6] Bascón L, Galvañ JI, López-Riquelme I, Navarro-Guillamón PJ, Morón JM, Llamas JA, Ballesteros M, Tejera-Vaquerizo A, Angulo AG, Guilabert A, Romaní J, en representación del grupo de estudio TCP (Tinea Capitis en Peluquería) (2023). Brote de dermatofitosis en región de cabeza y cuello asociadas al rasurado en peluquerías: estudio descriptivo multicéntrico de una serie de casos. Actas Dermosifiliogr.

